# The College of Nursing and the Minister of Health: Safeguarding the Public Interest

**Published:** 1919-10-11

**Authors:** M. S. Rundle

**Affiliations:** Secretary of the College of Nursing, Limited.


### The College of Nursing and the Minister of Health: Safeguarding the Public Interest.

The following is a copy of a letter which has been addressed to the Right Hon. C. Addison, M.B., M.P., Minister of Health, by the Secretary of the College of Nursing:

SIR,—The Council of the College of Nursing, Limited, notes that the Consultative Bodies under the Ministry of Health have now been announced, and they observe with great regret that the Consultative Council on Medical and Allied Services includes no trained nurses. The Council would very strongly urge upon the Minister of Health and his advisers that it is of paramount importance to the State that the nursing profession, so closely allied to the medical profession, and so vitally concerned with the health of the nation, should have adequate representation upon this body. It is stated that "it is intended when questions are under consideration that specially affect the nursing and midwifery services the Council shall be assisted by the appointment of a committee consisting partly of its own members, but also including practising nurses and midwives, and other persons not members of the Council who have devoted themselves specially to the study of nursing and midwifery questions."

This provision, whilst satisfactory so far as it goes, does not, in the opinion of the Council of the College of Nursing, meet the just claims of the nursing profession to a place on the Council for women able to speak authoritatively from the point of view of the trained nurse upon the many matters affecting the nursing profession, which must of necessity engage the attention of the Council on Medical and Allied Services. The Council of the College understands that the membership of the Consultative Council on general health questions is not yet completely settled, and on this Council also the nursing profession should, in their judgment, be strongly represented. In thus addressing you the Council of the College of Nursing, Limited, is confident that it expresses the opinion, not only of the 15,000 trained nurses upon its register, but of the nursing profession at large, and is confident that the claims on their behalf thus brought to your notice will receive your sympathetic consideration.

I am, Sir, yours faithfully,
(Signed) M. S. RUNDLE,
Secretary of the College of Nursing, Limited.

